# Molecular Identification, Mycelial Growth Kinetics, and Antimicrobial Potential of Newly Isolated Medicinal Mushroom *Fomitopsis pinicola* from Bulgaria

**DOI:** 10.3390/jof11100727

**Published:** 2025-10-10

**Authors:** Petya Stefanova, Anateya Georgieva, Mariya Brazkova, Radka Baldzhieva, Bogdan Goranov, Denica Blazheva, Anton Slavov, Galena Angelova

**Affiliations:** 1 Department of Microbiology and Biotechnology, University of Food Technologies, 26 Maritsa Blvd., 4002 Plovdiv, Bulgaria; hristina2001_7@abv.bg (A.G.); mbrazkova@uft-plovdiv.bg (M.B.); r_baldzhieva@uft-plovdiv.bg (R.B.); b_goranov@uft-plovdiv.bg (B.G.); g_angelova@uft-plovdiv.bg (G.A.); 2 Department of Organic Chemistry and Inorganic Chemistry, University of Food Technologies, 26 Maritsa Blvd., 4002 Plovdiv, Bulgaria; antons@uni-plovdiv.net

**Keywords:** *Fomitopsis pinicola*, medicinal mushroom, molecular identification, mycelial growth kinetics, antimicrobial activity

## Abstract

The present study is focused on a newly isolated *Fomitopsis* strain obtained from black pine (*Pinus nigra*) from the Sredna Gora Mountains, Bulgaria. Molecular identification, based on *ITS1-5.8S-ITS2* region sequencing, confirmed the strain as *Fomitopsis pinicola* with 99.84 BLAST percent identity. Phylogenetic analysis verified that the new fungal isolate belongs to the European *F. pinicola* clade. The morphological analysis of the strain revealed several distinctive structures that further support its identification. The influence of culture media composition on fungal development was evaluated by analyzing the mycelial growth kinetics using both the logistic growth model and the reversible autocatalytic model. Submerged cultivation was employed to produce fungal biomass, which was subsequently lyophilized and used for the assessment of the antimicrobial potential of the fungal strain. The results demonstrated notable antimicrobial effects against all tested bacterial strains. The most significant activity was observed for the aqueous extract against *Escherichia coli* and the hexane extract against *Salmonella enteritidis*, both with a minimum inhibitory concentration of 312.5 µg/mL. These findings highlight the promising potential of the newly isolated *F. pinicola* strain for future applications in the medical and pharmaceutical industries, particularly in developing drugs to combat multidrug resistance, based on the promising results of its water extracts.

## 1. Introduction

Higher fungi represent an exceptionally diverse group of eukaryotic organisms, exhibiting vast taxonomic, ecological, and physiological variability. Although an estimated 3.8 million fungal species exist in nature, recent studies suggest that less than 5% have been formally described and documented in databases such as Species Fungorum [1]. The division *Basidiomycota* is the second largest in the fungal kingdom, following *Ascomycota*, and currently includes over 31,000 identified species. However, the true diversity within *Basidiomycota* is likely much greater, as molecular ecology studies have uncovered a surprisingly high and previously unrecognized level of diversity both within this division and across the fungal kingdom as a whole [2,3]. Fungi belonging to *Basidiomycota* play vital roles in forest ecosystems and are increasingly recognized as valuable bioresources in biotechnology, medicine, and environmental applications. Their biological importance lies in their ability to degrade complex organic matter such as lignocellulose, contributing to nutrient cycling and promoting plant growth through mycorrhizal associations [4,5]. The class *Agaricomycetes*, belonging to the division *Basidiomycota* and the subkingdom *Dikarya*, includes fungi commonly known as higher fungi. These organisms are characterized by the formation of macroscopic fruiting bodies, or basidiocarps, which bear the spore-producing structures known as basidia [6]. Spores are released from these basidia as basidiospores, a distinguishing feature that sets them apart from fungi in other phyla [4,6,7].

The genus *Fomitopsis* (family *Polyporaceae*, order *Polyporales*) is highly diverse and includes numerous species found on both deciduous and coniferous trees. Members of this genus produce perennial, woody basidiocarps characterized by poroid hymenophores [8,9,10]. These fungi grow on a variety of gymnosperm and angiosperm hosts. As typical representatives of basidiomycete fungi, *Fomitopsis* species play a crucial role in natural ecosystems by contributing to the biodegradation of lignocellulosic material and the recycling of nutrients [11]. Because of their highly efficient enzymatic systems, fungi of the genus *Fomitopsis* are able to utilize wood mass into organic compounds, which are subsequently assimilated by other organisms. In this way, they enhance nutrient cycling and promote biodiversity within their habitats [12,13,14].

Recent research has increasingly focused on the wide array of bioactive compounds produced by fungi of the *Basidiomycota* division, including polysaccharides, glycoproteins, and triterpenoids. These compounds have demonstrated notable pharmacological properties, particularly antimicrobial, antitumor, and immunomodulatory activities [15,16]. In particular, the fruiting bodies of fungi from the genus *Fomitopsis* are rich in biologically active substances with significant medicinal potential. These include various enzymes, steroids, triterpenes, and both endopolysaccharides and exopolysaccharides. Collectively, these compounds contribute to a range of beneficial effects on human health, highlighting the genus *Fomitopsis* as a valuable source of natural therapeutic agents [17,18].

*Fomitopsis pinicola* (Schwartz: Fr.) Karst., commonly known as the red-belted conk, is a widespread species of brown-rot fungus found throughout the Northern Hemisphere. It colonizes a broad range of both gymnosperm and angiosperm trees [10,11,13]. The fruiting body is fan-shaped with a hard, woody texture, often reaching up to 40 cm in diameter. Its surface is typically glossy, with coloration ranging from reddish-brown to lighter shades, depending on the age of the specimen. This species plays a vital ecological role in forest ecosystems by contributing to nutrient cycling through the decomposition of dead tree trunks. Notably, the brown rot debris it produces can persist in the soil for extended periods before fully breaking down [10].

*F. pinicola* is well known for its significant medicinal and industrial potential. Traditionally used in Asian medicine, this species has demonstrated a broad spectrum of therapeutic properties, including anti-cancer, anti-inflammatory, anti-diabetic, anti-obesity, and immunomodulatory effects [19,20,21,22]. Chemical analyses of *F. pinicola* fruiting bodies have revealed a rich composition of biologically active compounds such as lanostane-type triterpenoids, triterpene glycosides, steroids, phenolic acids, β-glucans, chitin, and chitosan, many of which are known for their pharmacological benefits [5,16,18]. Extracts of *F. pinicola* have been shown to inhibit tumor cell growth and pathogens, including *Staphylococcus aureus*. The observed effects are thought to be the result of several mechanisms that include disruption of cell membranes, induction of apoptosis, and immunomodulation via β-glucan receptor pathways [22]. Importantly, studies have also indicated that *F. pinicola* extracts do not exhibit toxic effects on major organs such as liver, kidneys, heart, brain, or lungs, further supporting its potential as a safe therapeutic agent [17,18].

Beyond its medicinal value, *F. pinicola* also holds industrial relevance due to its production of key enzymes, including endoglucanase, laccase, cellobiohydrolase, thermostable xylanase, and β-1,4-glucosidase, which are utilized in sectors such as biofuel production, paper processing, and waste management [17,18].

As the world faces the impending threat of antibiotic resistance, the search for novel antimicrobial compounds has intensified. Fungi are a promising option with their extensive co-evolutionary history against microbial competition and potential for complex secondary metabolite production [22]. Microbial antagonism, which has been a subject of extensive research since the 20th century, has also substantiated the fact that fungi like *F. pinicola* are reservoirs of potent natural antibiotics [22,23].

Despite the numerous beneficial properties of *F. pinicola* fruiting bodies, achieving controlled in vitro cultivation remains a significant challenge in modern biotechnology. Wild higher fungi are a finite natural resource, and unregulated harvesting poses a serious threat to their reproduction and species diversity [24,25]. The application of controlled in vitro cultivation is an alternative and promising approach for producing fungal biomass rich in valuable bioactive compounds and extracellular secondary metabolites. This biotechnological strategy offers several advantages, including a significant reduction in cultivation time, consistent quality of bioactive metabolite production, lower volume requirements, minimal risk of contamination, and the possibility of cultivating wild fungal species under laboratory conditions. These benefits contribute to the conservation of natural fungal biodiversity by reducing pressure on wild populations [24,26].

Although numerous studies have examined the biological activities of *Fomitopsis pinicola*, most have focused on its fruiting body. In contrast, research on in vitro cultivation and the growth kinetics of *F. pinicola* on different nutrient media remains limited [25]. Moreover, the effects of controlled cultivation parameters on mycelial development, metabolite production in culture broth, and the antimicrobial activity of the mycelium are still not well understood. In light of these considerations, optimizing nutrient media composition and cultivation conditions is essential to enhance biomass yield and/or the production of target bioactive compounds. To address this, the present study is the first to investigate the in vitro mycelial growth kinetics of a newly isolated Bulgarian strain of *F. pinicola* across various culture media, along with an evaluation of its antimicrobial potential.

## 2. Materials and Methods

### 2.1. Fungal Isolation, In Vitro Cultivation, and Morphological Characterization

The fruiting bodies of the newly isolated strain were collected from black pine (*Pinus nigra*) from the Sredna Gora Mountains, Bulgaria (42.419722°, 25.158864° in May 2024). The mushroom was collected from a public area where it is not classified as a protected species; therefore, no special permissions were necessary for collection. After the collection, undamaged portions of the fruiting body were selected, cut, and rinsed with sterile distilled water. Surface disinfection followed the protocol of Angelova et al. [27]. It includes treatment with 70% ethanol, then 2% sodium hypochlorite (NaClO), and a final rinse with sterile distilled water. Disinfected samples were cut into 5 by 5 mm pieces using a sterile scalpel and aseptically transferred onto Rose Bengal Chloramphenicol Agar (RBCA) (HiMedia Laboratories GmbH, Modautal, Germany). The plates were incubated in darkness at 28 °C for 8 days. The obtained unidentified fungal colonies were isolated and purified through repeated subculturing on fresh medium. The resulting pure fungal isolate was cultivated on Mushroom Complete Medium (MCM), composed of (g/L) glucose—20.0, KH_2_PO_4_—0.5, K_2_HPO_4_—1.0, MgSO_4_—0.5, peptone—2.0, yeast extract—2.0, agar—2.0, at pH 5.5–6.0. Fully developed cultures were stored at 4 °C for subsequent morphological and molecular analyses.

Macroscopic features of the fungal isolate (including texture, size, and coloration of the top of the colony surface and the reverse side of the plate) were monitored daily. Microscopic characterization was conducted using an Olympus CX33 biological microscope (Olympus, Tokyo, Japan), following sample preparation by mounting mycelium in water or using the Scotch tape imprint method.

### 2.2. Molecular Identification by ITS1-5.8S-ITS2 rRNA Gene Sequence Analysis

Before DNA extraction, the fungal isolate was cultured on MCM agar plates for 7 days. Approximately 100–300 mg of mycelium was scraped using a sterile spatula and transferred into a 2 mL microcentrifuge tube. Total DNA was extracted using a modified CTAB method, according to Stefanova et al. [28]. The quality and concentration of the DNA extracts were assessed by determination of their absorbance at 260 nm and 280 nm (Shimadzu UV-VIS, Shimadzu Corporation, Kyoto, Japan). The ITS-5.8S-ITS2 region was amplified by forward primer ITS 4 (5′-TCCTCCGCTTATTGATATGC-3′) and reverse primer ITS 5 (5′-GGAAGTAAAAGTGCTAACAAGG-3′) [7], obtained from Metabion (Martinsried, Germany). The PCR reaction mix contained 1 μL of DNA (50 ng/μL), 0.5 μM of each primer, and 8 μL of Red-Taq DNA Polymerase Master Mix (Canvax Biotech, S.L., Valladolid, Spain) in a total volume of 20 μL. The amplification was carried out in a PCR 2720 Thermal Cycler (Applied Biosystems, Carlsbad, CA, USA) using the following program: initial denaturation at 95 °C for 10 min, followed by 35 cycles of 1 min at 95 °C, 1 min at 52 °C, and 1 min at 72 °C, and final extension at 72 °C for 7 min. The PCR product was visualized in a 1% agarose gel stained with SafeView (NBS Biologicals, Huntingdon, UK) at 100 V for 50 min using VWR Mini Electrophoresis System (VWR, Darmstadt, Germany) and MiniBis Pro (DNR Bio-Imaging Systems, Jerusalem, Israel) for gel visualization.

Finally, the PCR product was cut out from the gel and purified with Clean-Easy™ Agarose Purification Kit (Canvax Biotech, S.L., Valladolid, Spain). Sequencing of the PCR product was performed by Microsynth Seqlab (Göttingen, Germany). The resulting sequence was analyzed using the BLAST algorithm (v. 2.17.0) [29] and compared against nucleotide sequences available in the GenBank database [30]. The *ITS1-5.8S-ITS2* rRNA gene sequence of the fungal isolate was deposited in the GenBank database, and an accession number was assigned.

### 2.3. Phylogenetic Analysis

The phylogenetic analysis was conducted using the closest matched ITS sequences from the GenBank database [30] and the ITS sequences of five other species of the genus *Fomitopsis*. *Niveoporofomes spraguei* (GenBank Accession No. MH114658.1) was used as an outgroup taxon. CLC Genomics Workbench 20.0 [31] was used to align the sequences and build the phylogenetic tree by means of the Unweighted Pair Group Method using the Arithmetic Average (UPGMA) clustering algorithm [32]. The nucleotide distance was evaluated by the Jukes–Cantor distance model, and standard bootstrap analysis with 1000 replicates was performed.

### 2.4. Cultivation Procedure and Kinetic Modeling of the Process

Cultivation of the strain was carried out on Petri dishes containing twelve distinct culture media: Czapek Dox (CD), glucose-peptone (GP), Hennerberg, Hopkins, Leonian, MCM, malt extract agar (MEA), malt yeast extract (MYE), potato dextrose agar (PDA), yeast extract agar (YEA), yeast glucose chloramphenicol (YGC), and yeast extract malt extract agar (YMA) [33].

The composition of the media is presented in Table 1.

Inoculation of the media was performed by transferring agar disks (d = 10 mm) of a fully grown culture. Surface cultivation was conducted under static conditions in a thermostat at 25 °C for 14 days. Colony diameter was measured daily, and growth density was recorded. Each culture medium was inoculated in five replicates (*n* = 5).

The resulting data were then utilized for the modeling of the growth kinetics by applying the logistic curve model (Equation (1)) and the reversible autocatalytic growth model (Equation (2)) [34,35].

(1) $$\frac{dD}{d\tau }=\mu_{max}D-\delta D^{2}$$

(2) $$\frac{dD}{d\tau }=k_{1}S_{0}^{\prime }D-\frac{k_{1}S_{0}^{\prime }}{D}D^{2}\to D=\frac{D_{0}S_{0}^{\prime }\frac{K}{1+K}}{D_{0}-\left(S_{0}^{\prime }\frac{K}{1+K}-D_{0}\right)e^{-k_{1}S_{0}^{\prime }\tau }}\;D_{m}=S_{0}^{\prime }\frac{K}{1+K}$$

where parameters *μ_max_* (specific growth rate, d^−1^), *D_0_* (initial diameter of the mycelium, mm), *D* (current diameter of the mycelium, mm), *D_m_* (maximal diameter of the mycelium, mm), *k_1_* (biomass yield rate constant, d^−1^), *S_0_′* (initial substrate quantity in cell units described with the diameter of the mycelia, mm), *K/1 + K* (substrate utilization factor), and *τ* (cultivation time, day) were identified as parameters of interest.

The logistic curve model was solved using the fourth-order Runge–Kutta method. Model parameter identification was performed by minimizing the sum of squared differences between experimental and model data using Excel’s Solver function [36,37].

### 2.5. Determination of Antimicrobial Activity

#### 2.5.1. Submerged Cultivation of *Fomitopsis pinicola*

The submerged cultivation of the pure culture was conducted in 500 mL Erlenmey-er’s flasks containing 100 mL MCM. The flasks were placed on a rotary shaker at 220 rpm and 25 °C for 7 days in the absence of light. At the end of the cultivation process, the biomass was separated from the medium by filtering on a Buhner’s funnel. The mycelial biomass was washed with distilled water, lyophilized, and then ground into a fine powder.

#### 2.5.2. Preparation of Extracts from Mycelial Biomass

The preparation of extracts from fungal biomass was conducted according to Angelova et al. [38] with some modifications. In brief: 0.5 ± 0.05 g of lyophilized mycelial biomass was mixed with 20 mL of each solvent (water, methanol, ethanol, butanol, ethyl acetate, methylene chloride, and hexane). The mixtures were incubated on a laboratory shaker at 150 rpm for 24 h at 25 °C. Following incubation, the samples were centrifuged at 6000 rpm for 15 min at 4 °C, and the resulting supernatants were collected and stored at −18 °C. A second extraction was then performed by adding a fresh portion of each solvent to the residual biomass under the same conditions, followed by centrifugation and collection of the supernatants. This extraction procedure was conducted in triplicate. In addition, hot water extraction was performed as follows: 0.5 ± 0.05 g of lyophilized mycelial biomass was mixed with 60 mL of hot water, and the mixture was incubated on a laboratory shaker at 150 rpm for 8 h at 80 °C. The combined extracts were evaporated under vacuum at 40 °C until completely dry. The dried extracts were then dissolved in dimethyl sulfoxide (DMSO) to a final concentration of 10 mg dry weight (DW)/mL and were sterilized through membrane filtration, using sterile 0.45 µm PTFE syringe filters (Merck, Darmstadt, Germany).

#### 2.5.3. Determination of the Minimum Inhibitory Concentration (MIC) of *Fomitopsis pinicola* extracts

The following microbial strains were used for the determination of MIC of the obtained mycelium extracts: *Escherichia coli* ATCC 8739, *Salmonella enterica* ssp. enterica ser. enetritidis ATCC 13076, *Staphylococcus aureus* ATCC 25923, *Pseudomonas aeruginosa* ATCC 9027, *Listeria monocytogenes* ATCC 8787, *Klebsiella pneumoniae* ATCC 13883, *Bacillus subtilis* ATCC 6633, *Bacillus cereus* ATCC 11778. The bacterial strains were provided from the collection of the Department of Microbiology and Biotechnology at the University of Food Technologies, Plovdiv, Bulgaria. The strains were grown on LBG agar with the following composition (g/L): peptone from casein—10.0; yeast extract—5.0; glucose—10.0; NaCl—10.0; agar—15.0, and pH prior to sterilization 7.0.

The minimum inhibitory concentration for each extract was evaluated according to the CLSI method [39]. The extracts were subjected to serial two-fold dilutions in Mueller-Hinton broth (Merck, Germany) using a 96-well microtiter plate. Then, each well was inoculated with a microbial suspension with a concentration of 5.10^5^ CFU/mL. After mixing, the plates were incubated at 37 °C for 18 h. The MIC is the lowest concentration of extract that completely inhibited the growth of the test microorganism.

### 2.6. Statistical Analysis

All cultivations were performed with five replicates (*n* = 5). The results obtained are presented as the arithmetic mean of the five replicates, with the standard deviation (SD) indicated as a measure of the variability. The statistical significance was determined by the analysis of variance (ANOVA and Tukey’s HSD test); the value of *p* < 0.05 indicated a statistical difference [40]. All statistical analyses were conducted with the software Statgraphics (Version 18.1.12), Centurion 18 Statistical Software.

## 3. Results and Discussion

### 3.1. Morphological Characterization

**Figure 1:**
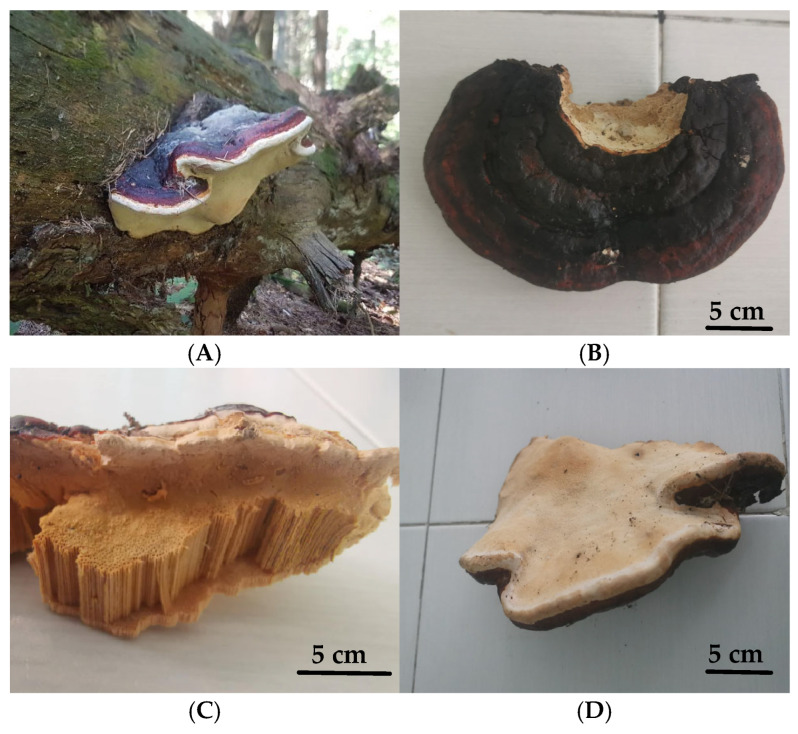
*Fomitopsis pinicola* fruiting body: basidiocarp in situ (**A**); upper surface (**B**); inner part (**C**); lower surface (**D**). Scale bar = 5 cm (**B**–**D**).

The basidiocarp collected from a dead black pine tree (Figure 1A) was used to establish a pure mycelial culture by in vitro cultivation on MCM agar. Preliminary examination of the morphology of the fruiting body and macroscopic and microscopic characteristics of the fungal culture indicated its affiliation with the genus *Fomitopsis.* The fruiting body had a woody texture and displayed a flattened, semi-spherical morphology with a smooth surface and an approximate width of about 30 cm.

The upper surface was dark reddish-brown, characterized by thin margins and distinct milky-white margin bands (Figure 1A). The lower surface displayed a milky-yellow to light yellow–brown coloring (Figure 1D). The inner part had a clearly visible small tube-like structure, which is typical for the mushrooms belonging to the *Polyporales* genus (Figure 1C). These tubes are densely arranged and oriented vertically, forming a porous hymenial surface on the inner part of the fruiting body.

**Figure 2:**
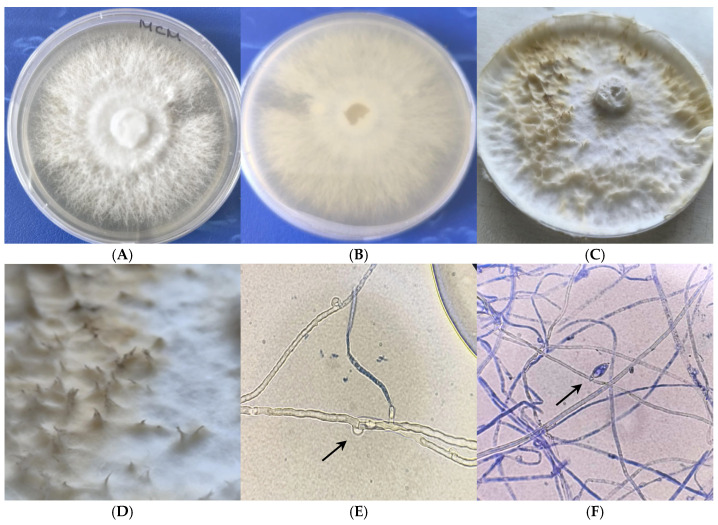
Colony morphology of fungal isolate on MCM: upper surface (**A**); lower surface (**B**); in vitro teleomorph formation (**C**,**D**); Mycelial structure: septate branched hyphae with clamps (100×) (**E**); chlamydospores (100×) (**F**).

The mycelial culture displayed a uniform radial growth pattern over the agar surface, producing white colonies characterized by well-developed aerial mycelium (Figure 2A). No pigmentation was observed on the reverse surface (Figure 2B). A faint mushroom-like odor was detectable. After about a month of incubation, the obverse surface of the colony developed slight browning, while the reverse side of the medium displayed a subtle yellow discoloration (Figure 2C).

In vitro primordia formation was observed after two months of cultivation on MCM agar at 25 °C (Figure 2C,D), likely as a result of culture aging and progressive nutrient depletion. The presence of primordia suggested the beginning of the sexual cycle, which is necessary for the formation of a mature teleomorph, but no such teleomorph or basidiospores were observed.

The mycelium is characterized by having hyaline, thin-walled, septate hyphae (Figure 2D), with rounded clamp connections (Figure 2E). Clamp connections are consistently present at most septa and represent a diagnostic morphological feature of the *Basidiomycota*. Lemon-shaped chlamydospores were observed in the culture after a month of cultivation on MCM agar at 25 °C (Figure 2F). Fungal identification is primarily based on phenotypic characteristics; however, due to the morphological similarity of *Fomitopsis pinicola* to *Ganoderma lingzhi* and other species within the genus *Fomitopsis*, molecular techniques are required to accurately confirm species identity and phylogenetic affiliation [10].

### 3.2. Molecular Identification and Phylogenetic Analysis

Molecular identification of the 7-day mycelial culture was performed by the amplification of the *ITS1-5.8S-ITS2* region, and the obtained PCR product was subjected to sequence analysis. The resulting sequence was analyzed using the BLAST algorithm and compared to the nucleotide sequences in the GenBank database [30]. The strain was identified as *Fomitopsis pinicola* with 99.84 BLAST percent identity. The *ITS1-5.8S-ITS2* rRNA gene sequence of the newly isolated *F. pinicola* was deposited in GenBank under accession number PX069912.

**Figure 3:**
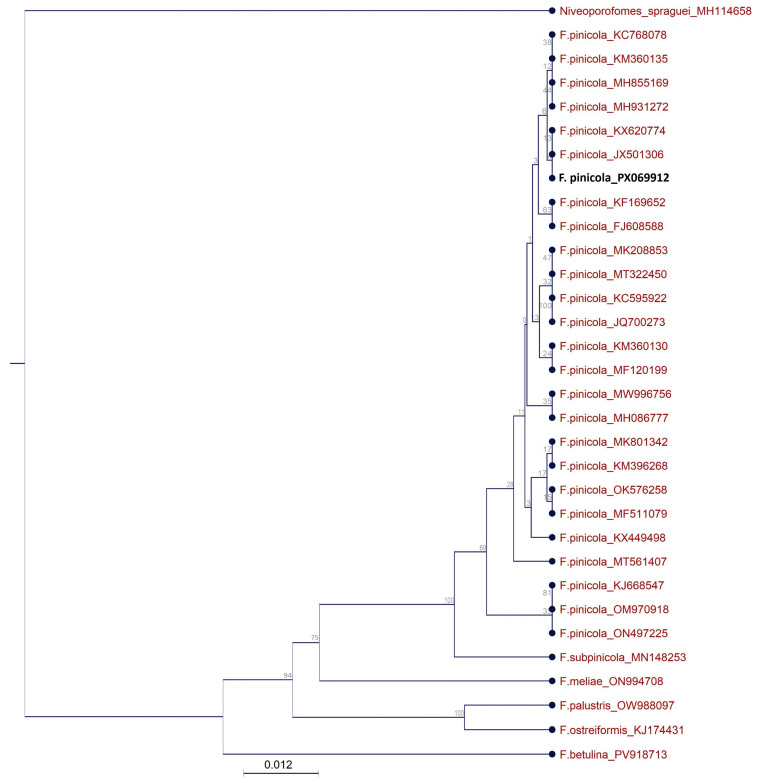
Phylogenetic relationship between *F. pinicola* PX069912, closely related *F. pinicola* strains, and some other species of genus *Fomitopsis* based on partial sequences analysis of the *ITS1-5.8S-ITS2* region.

The phylogenetic relationships among *F. pinicola* PX069912, twenty-five closely related *F. pinicola* strains, five other species within the genus *Fomitopsis*, and *Niveoporofomes spraguei* (used as an outgroup taxon) were examined based on partial sequence analysis of the *ITS1-5.8S-ITS2* region (Figure 3).

The phylogenetic analysis clearly demonstrated that the newly isolated fungal strain clusters within the same clade as all other European *F. pinicola* strains. These findings are consistent with the results of Gáper et al. (2025), who also reported a lack of genetic divergence among the examined *F. pinicola* strains [41]. Interestingly, *F. pinicola* strains originating from geographically distant regions, such as KJ668547 (South Korea), ON497225 (China), and OM970918 (China), formed a separate clade, indicating a more distant phylogenetic relationship compared to the European isolates. The result is supported by Dresch et al. (2015), who reported very little sequence divergence among European *F. pinicola* strains, but observed some sequence diversity between European and Asian strains [42]. All other species of the genus *Fomitopsis* were grouped into a distinct clade. Notably, *F. subpinicola* MN148253 was the most closely related to the examined *F. pinicola* strains among the other *Fomitopsis* species. This observation aligns with findings by Liu et al. (2022), who reported that *F. pinicola* strains are more closely related to *F. subpinicola* than to *F. betulina* [11]. *Fomitopsis meliae* is also closely related to the *F. pinicola* clade, which is consistent with the findings of Dresch et al. [42].

### 3.3. Kinetic Modeling of the Process

**Figure 4:**
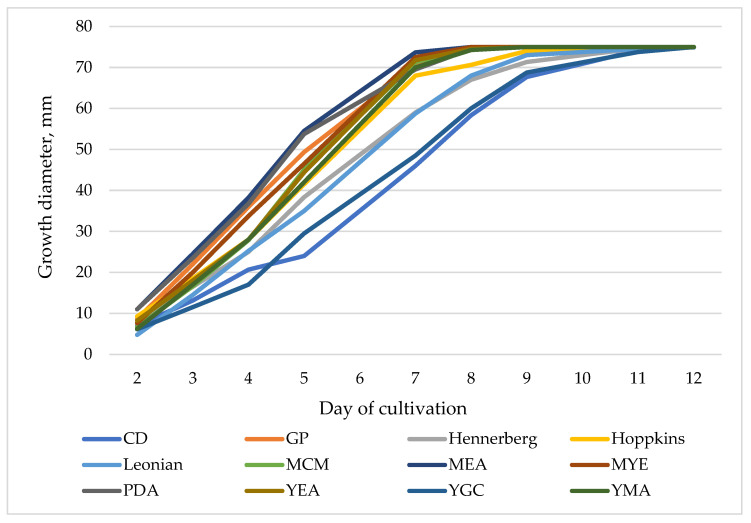
Growth dynamics of *F. pinicola* on different culture media.

**Figure 5:**
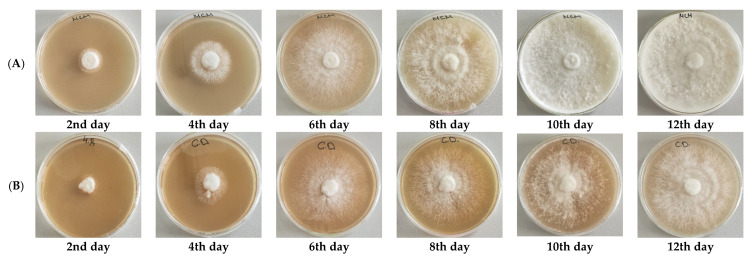
Mycelial growth of *F. pinicola* on MCM (**A**) and CD (**B**).

Since controlled biotechnological cultivation offers a promising alternative for producing fungal biomass enriched with valuable bioactive components and extracellular secondary metabolites, it is important to investigate the potential of the newly isolated *F. pinicola* strain for in vitro cultivation. Therefore, the fungus was cultured on twelve different media (CD, GP, Hennerberg, Hopkins, Leonian, MCM, MEA, MYE, PDA, YEA, YGC, and YMA) to assess their influence on the growth of the strain. The primary growth parameter evaluated was the increasing diameter of the mycelium, which served as an indicator of the growth capacity of *F. pinicola* on each medium. Colony diameter was monitored over a 12-day cultivation period at 25 °C, and the experimental results are presented in Figure 4 and Figure 5.

As shown in Figure 4, the growth of the strain varies in intensity across the tested synthetic media, with a maximum colony diameter of 75 mm observed. The most rapid growth occurs on GP, MCM, MEA, MYE, PDA, YEA, and YMA media, where the maximum colony diameter is reached by the 9^th^ day of cultivation. On MCM medium, *F. pinicola* produces well-defined radial colonies characterized by dense, white aerial mycelium with a fine, cotton-like texture. At early stages, the colonies display a fluffy or woolly appearance, subsequently becoming progressively denser and more compact. No pigment production is observed throughout the developmental process (Figure 5A).

In contrast, on the remaining media, maximal diameter was achieved by the 12th day, indicating slower growth rates. The slowest radial growth and lowest mycelial density of *F. pinicola* observed on CD medium, compared to all other tested media, could be attributed to the use of sucrose as the carbon source (Figure 4 and Figure 5B). This finding aligns with previous studies, which indicate that sucrose is more difficult for many basidiomycete fungi to absorb and metabolize than glucose. Glucose, by contrast, is more readily assimilated and supports more robust fungal growth [25,43]. Experimental data from the cultivation of the strain on CD, Hennerberg, and Hopkins media show significantly lower density of the fungal colony compared to other synthetic media (Figure 4). This reduced growth is likely due to the presence of inorganic nitrogen in the form of nitrate ions in these media. In contrast, the remaining nutrient media contain organic nitrogen sources such as malt extract, yeast extract, or peptone. The superior absorption and utilization of organic nitrogen by many basidiomycete fungi have been well-documented, with studies reporting that organic sources support faster development and denser mycelial growth compared to nitrate-based inorganic sources [44,45,46].

A comprehensive understanding of process kinetics is crucial for the successful industrial cultivation of *F. pinicola*. One of the primary factors influencing the strain’s kinetic behavior is the composition of the culture medium. A combination of the logistic growth model and the reversible autocatalytic model was applied to assess the cultivation kinetics. Model parameter identification and estimation of key kinetic constants were carried out, with the results summarized in Table 2.

The data presented in Table 2 shows that both models are characterized by high correlation coefficients, ranging from 0.9001 to 0.9988, indicating a strong relation with the experimental results. According to the logistic growth model, the highest maximum specific growth rates (*µ_max_*) were observed on MCM, MEA, YMA, and MYE media, with values of 0.925 ± 0.031 d^−1^, 0.903 ± 0.010 d^−1^, 0.901 ± 0.032 d^−1^, and 0.900 ± 0.010 d^−1^, respectively. Other synthetic media that supported relatively high growth rates of *F. pinicola* included GP, Hennerberg, Hopkins, Leonian, PDA, and YEA, with *µ_max_* values of 0.879 ± 0.035 d^−1^, 0.710 ± 0.054 d^−1^, 0.802 ± 0.067 d^−1^, 0.777 ± 0.061 d^−1^, 0.845 ± 0.020 d^−1^, and 0.870 ± 0.016 d^−1^, respectively. The lowest *µ_max_* values were recorded on CD and YGC media (0.605 ± 0.075 d^−1^ and 0.649 ± 0.043 d^−1^, respectively), likely due to the specific composition of these media. Of particular interest in the kinetic analysis is the growth inhibition coefficient (*δ*), which varied across the tested media from 0.0079 ± 0.0012 mm·d^−1^ to 0.0134 ± 0.0024 mm·d^−1^. These values, being significantly lower than 1, indicate the absence of inhibitory effects in the media used.

As shown in Table 2, the biomass formation rate constant (*k_1_*) is lowest for the CD medium, with a value of 0.0045 ± 0.0005 d^−1^, which is significantly lower than those observed for the other media. This finding, consistent with the results from the logistic growth model, suggests that the composition of the CD medium is clearly suboptimal for the development of the strain. According to the reversible autocatalytic growth model, the highest *k_1_* values, indicating the most favorable conditions for biomass formation, were observed on MCM, MEA, and MYE media, with rate constants of 0.0093 ± 0.0002 d^−1^, 0.0095 ± 0.0003 d^−1^, and 0.0094 ± 0.0002 d^−1^, respectively. They were followed by PDA, YEA, YGC, YMA, GP, Leonian, Hennerberg, and Hopkins media, where *k_1_* values ranged from 0.0060 ± 0.0017 d^−1^ to 0.0088 ± 0.0001 d^−1^ (Table 2).

In the reversible autocatalytic growth model, a key quantity of interest is the substrate utilization factor (*K/(1 + K*)). The closer this parameter is to 1, the more optimal the medium composition and growth conditions are for culture development. As shown in Table 2, the highest value of this coefficient was recorded for GP medium (0.9055 ± 0.0011), suggesting a relatively well-balanced composition and more efficient substrate assimilation. In contrast, the lowest value was observed for the CD medium (0.6733 ± 0.0211). For the remaining media, *K/(1 + K)* values ranged from 0.7273 ± 0.0067 to 0.8444 ± 0.0021, depending on the formulation. These findings highlight the need to optimize both the composition of the nutrient media and the cultivation conditions to improve substrate utilization efficiency.

Statistical analysis of the kinetic parameters was performed using Tukey’s HSD test to identify significant differences. Regarding the maximum specific growth rate (*µ_max_*), the following statistically significant differences were observed: (1) for CD, *µ_max_* differed significantly from those measured on the GP, Hopkins, Leonian, MCM, MEA, MYE, PDA, YEA, and YMA media; (2) for GP, a significant difference in *µ_max_* was found compared to the YGC and CD media; (3) for Hennerberg, *µ_max_* differed significantly from that on MCM, MEA, MYE, PDA, YEA, and YMA media; (4) for Hopkins, a significant difference in *µ_max_* was observed only with the YGC medium; (5) for Leonian, *µ_max_* differed significantly from that on MCM and YGC media; (6) for MCM, the only statistically significant difference in *µ_max_* was with the YGC medium. A similar trend was observed for the MEA, MYE, PDA, and YEA media, where *µ_max_* values were statistically different only from that on YGC medium.

In the context of the substrate utilization factor (*K/(1 + K)*) from the reversible autocatalytic growth model, the following statistically significant differences were observed: (1) for CD and GP, the *K/(1 + K)* values differed significantly from those of all other media; (2) for Hennerberg, significant differences in *K/(1 + K)* were found compared to CD, GP, Hopkins, Leonian, MEA, and MYE media; (3) the Hopkins medium showed statistically significant differences in *K/(1 + K)* compared to YEA, YGC, YMA, MCM, CD, and GP media; (4) for Leonian, the substrate utilization factor was significantly different from that of YEA, YGC, YMA, CD, and GP media; (5) the MCM showed statistically significant differences in *K/(1 + K)* compared to YEA, YGC, CD, and GP media; (6) for MEA, significant differences were observed with YEA, YGC, YMA, CD, and GP media; (7) the MYE medium also differed significantly from YEA, YGC, YMA, CD, and GP media; (8) for PDA, statistically significant differences in the substrate utilization factor were observed with YEA, YGC, CD, and GP media; (9) lastly, for YEA, the *K/(1 + K)* value differed significantly from those of PDA, MYE, MCM, Leonian, Hopkins, CD, and GP media. These results indicate that media such as CD and GP stand out with distinctly different substrate utilization efficiency, while others cluster more closely together, depending on their composition and compatibility with *F. pinicola* growth.

With respect to the biomass formation rate constant *(k_1_*), statistically significant differences were observed between CD and the Leonian, MCM, MEA, MYE, PDA, YEA, YGC, and YMA media. For GP, *k_1_* differed significantly from the values recorded for MCM, MEA, and MYE. In the case of Hennerberg, significant differences in *k_1_* were found compared to MCM, MEA, MYE, and PDA media. Lastly, for Hopkins, statistically significant differences in *k_1_* were observed in comparison with MCM, MEA, MYE, PDA, YEA, YGC, and YMA.

Based on the results of the kinetic analysis, it can be concluded that the MCM, MEA, MYE, PDA, YEA, and YMA media are the most suitable candidates for developing an optimized synthetic nutrient medium for the cultivation of *F. pinicola*. These findings are consistent with previous studies, which have also identified MEA [47,48,49,50], MCM [33,51,52], PDA [33,50,53], MYE [54], YEA [55], and YMA [52,55] as favorable media for the cultivation of basidiomycete fungi.

### 3.4. Antimicrobial Activity of F. pinicola Extracts

In the investigation of the antimicrobial activity of the newly isolated *F. pinicola*, eight pathogenic bacteria, including both Gram-positive and Gram-negative, were used. The resistance of the pathogens to the obtained extracts with different solvents—water, hot water, ethanol, methanol, butanol, hexane, ethyl acetate, and methylene chloride—was studied. The results, presented in Table 3, show that all extracts of *F. pinicola*, obtained with the different solvents, exhibit antimicrobial activity towards the used test microorganisms. The most notable results in terms of minimum inhibitory concentration (MIC = 312.5 µg/mL) were observed for the extracts obtained with hot and cold water against *Escherichia coli* and hexane against *Salmonella enterica*.

The extract obtained with methylene chloride showed the strongest activity against *Listeria monocytogenes*. All extracts, except the aqueous ones, exhibited strong activity against *Bacillus subtilis*, while the other *Bacillus* representative—*Bacillus cereus*, was more resistant. It was more sensitive when exposed to the methanol and ethyl acetate extracts. *Pseudomonas aeruginosa* was most resistant to the effects of the ethyl acetate, followed by the hexane extract, and the extract obtained with hot water. *Klebsiella pneumoniae* showed good sensitivity against all extracts, and was slightly more resistant to the hot water extract. The weakest inhibitory effect for *E. coli* was expressed by the ethyl acetate and hexane extracts, and for *S. enterica*—by the butanol and ethyl acetate extracts.

Several authors report antimicrobial activity of different compounds, extracted from the fruiting bodies of *F. pinicola,* namely terpenoids and steroids [56,57]. More recently, Dresch et al. [42] investigated the activity of an ethanol extract of the fruiting body of *F. pinicola* and observed MICs between 31 and 125 μg/mL against *B. subtilis* and of 31–500 μg/mL against *S. aureus*. Karaca et al. [58] reported MIC of 312.5 µg/mL for an ethanol extract against two MRSA strains. Considering the differences in extracting procedures our results are in line with some of the strains in this study. Moreover, unlike the strains tested by Dresch et al. [42], our strain exhibited activity against *E. coli* and *P. aeruginosa* in concentrations below 1000 µg/mL. Pala et al. [59] also reported antimicrobial activity of fruiting body ethyl acetate and methanol extracts against *B. subtilis*, *E. coli*, *S. aureus*, *K. pneumoniae*, *P. aeruginosa*, and *Proteus vulgaris*. In comparison, their fungal strain did not exhibit an antimicrobial effect on the test strains after extraction with water, while our strain produces active cold and hot water extracts. Huguet et al. [60] studied the effect of French mushrooms, including *F. pinicola,* against pathogenic bacteria and multidrug-resistant clinical isolates of *E. coli* and *S. aureus.* They found that their methanol and ethyl acetate *F. pinicola* extracts contained the highest number of antimicrobial compounds and were active against all wild-type and multidrug-resistant bacteria included in the experiment. The investigations, reported in the available literature, mostly study the antimicrobial effect of organic solvent extracts. Bragina et al. [61] compared the activity of ethanol and hot water extracts and found that the organic solvent extracts have more promising inhibitory effect on three Gram-negative bacteria. This supports our findings regarding the antibacterial activity against *K. pneumoniae*. A comparison of the antibacterial activity of our extracts within the context of other medicinal mushrooms reveals the promising potential of our strain of *F. pinicola*. Alves et al. [62] studied methanol/water extracts of 13 mushroom species from Portugal against 16 Gram-negative and Gram-positive bacteria. The reported MICs are much higher than the ones determined by us (from 5000 to > 20,000 µg/mL). Three types of extracts—chloroform, 70% ethanol and hot water—form three mushrooms (two *Trametes* ssp. and one *Mircoporus* spp.) which demonstrated MICs in the range of 670 to 2000 µg/mL against *E. coli, K. pneumoniae, P. aeruginosa*, MRSA and *S. aureus* [63]. Gebreyohannes et al. [63] found that the water extracts performed better than those obtained with less polar solvents. The methodology they used to determine the MICs was different than ours, but the reported values are in the same range. These findings highlight the medicinal potential of *F. pinicola* and the importance of the development of a controlled method for the production of active extracts. All solvents included in this investigation showed significant antimicrobial activity. Considering the harmful effect some of them have on the environment and their price in the ultra-pure form suitable for human medical preparations, the results for the water extracts are very promising for future drug development in the context of multidrug resistance.

Building on the promising results obtained, future work will focus on the isolation and characterization of specific bioactive components, such as polysaccharides, proteins, phenolic compounds, and other metabolites, and further evaluation of their individual contributions to the observed biological activities. We consider the present study a preliminary yet essential step toward these more comprehensive biochemical and pharmacological investigations. Planned follow-up studies will include fractionation of the raw biomass to isolate distinct compound classes, quantitative and qualitative analysis of key extract constituents (e.g., total protein, polysaccharide, and phenolic content), structural characterization of bioactive molecules (e.g., β-glucans, peptides, phenolics), and correlation of compound profiles with specific antimicrobial or antioxidant effects. In parallel, cultivation conditions will be optimized to enhance the yield of targeted bioactive compounds.

## 4. Conclusions

Controlled in vitro cultivation offers a promising alternative for producing fungal biomass, enriched with valuable bioactive components and extracellular secondary metabolites. This study is the first to investigate the mycelial growth kinetics of a newly isolated *F. pinicola* strain on various synthetic media. Optimizing nutrient composition is essential to enhance biomass yield and/or the production of target bioactive compounds. Growth kinetics were evaluated using both the logistic curve and reversible autocatalytic growth models, enabling the selection of the most suitable candidates for developing optimized synthetic media for *F. pinicola* cultivation. Finally, the antimicrobial potential of the fungal strain was assessed and the results demonstrated notable antimicrobial effects against all tested bacterial strains, especially the aqueous extract against *Escherichia coli* and the hexane extract against *Salmonella enteritidis*.

This study represents an important foundation for more in-depth biochemical and pharmacological research. Encouraged by the promising findings, future work will aim to isolate and structurally characterize key bioactive compounds, such as polysaccharides, proteins, phenolics, and other metabolites, and to determine their specific roles in the observed biological effects. Overall, this research highlights the significant potential of the newly isolated medicinal mushroom *F. pinicola* for future applications in the medical and pharmaceutical industries.

## Figures and Tables

**Table 1:** Composition of the culture media used for cultivation.

Component, g/L	Culture Media											
	CD	GP	Hennerberg	Hopkins	Leonian	MCM	MEA	MYE	PDA	YEA	YGC	YMA
Glucose	-	10.0	50.0	10.0	25.0	20.0	-	10.0	20.0	10.0	20.0	10.0
Sucrose	30	-	-	-	-	-	-	-	-	-	-	-
Peptone	-	10.0	-	-	-	2.0	3.0	-	-	-	-	5.0
Yeast extract	-	10.0	-	-	-	2.0	-	5.0	-	5.0	5.0	3.0
Malt extract	-	15.0	-	-	-	-	30.0	3.0	-	-	-	3.0
Potato extract	-	-	-	-	-	-	-	-	4.0	-	-	-
NaNO_3_	3.0	-	2.0	-	-	-	-	-	-	-	-	-
MgSO_4_.7H_2_O	0.5	-	0.5	0.5	0.5	0.5	-	-	-	-	-	-
KCl	0.5	-	-	-	-	-	-	-	-	-	-	-
FeSO_4_.7H_2_O	0.01	-	-	-	0.02	-	-	-	-	-	-	-
CaCl_2_.2H_2_O	-	-	0.1	-	-	-	-	-	-	-	-	-
ZnSO_4_.7H_2_O	-	-	-	-	-	-	-	-	-	-	-	-
MnSO_4_.5H_2_O	-	-	-	-	0.01	-	-	-	-	-	-	-
K_2_HPO_4_	1.0	-	-	-	-	1.0	-	-	-	-	-	-
KH_2_PO_4_	1.0	-	1.0	0.1	1.0	0.5	-	-	-	-	-	-
KNO_3_	-	-	2.0	2.0	-	-	-	-	-	-	-	-
Chloramphenicol	-	-	-	-	-	-	-	-	-	-	0.1	-
Agar	20	20	20	20	20	20	20	20	20	20	20	20

**Table 2:** Kinetic parameters of the *F. pinicola* cultivation models on different synthetic culture media.

Medium	Logistic Curve Model			Reversible Autocatalytic Growth			
	µ_max_, d^−1^	δ, mm.d^−1^	R^2^	K_1_, d^−1^	S 0′	K/1 + K	R^2^
CD	0.605 ± 0.075	0.0079 ± 0.0012	0.9946	0.0045 ± 0.0005	86 ± 0.2	0.6733 ± 0.0211	0.9001
GP	0.879 ± 0.035	0.0097 ± 0.0044	0.9963	0.0065 ± 0.0021	88 ± 2.0	0.9055 ± 0.0011	0.9639
Hennerberg	0.710 ± 0.054	0.0082 ± 0.0017	0.9988	0.0064 ± 0.0003	101 ± 1.0	0.7658 ± 0.0229	0.9983
Hopkins	0.802 ± 0.067	0.0106 ± 0.0010	0.9974	0.0060 ± 0.0017	104 ± 2.0	0.8298 ± 0.0142	0.9974
Leonian	0.777 ± 0.061	0.0106 ± 0.0011	0.9952	0.0074 ± 0.0004	90 ± 0.3	0.8444 ± 0.0021	0.9971
MCM	0.925 ± 0.031	0.0134 ± 0.0024	0.9972	0.0093 ± 0.0002	96 ± 0.3	0.7967 ± 0.0093	0.9874
MEA	0.903 ± 0.010	0.0127 ± 0.0013	0.9975	0.0095 ± 0.0003	92 ± 0.4	0.8193 ± 0.0044	0.9975
MYE	0.900 ± 0.010	0.0123 ± 0.0011	0.9949	0.0094 ± 0.0002	91 ± 0.1	0.8321 ± 0.0108	0.9951
PDA	0.845 ± 0.020	0.0112 ± 0.0003	0.9981	0.0088 ± 0.0002	94 ± 3.0	0.8034 ± 0.0213	0.9982
YEA	0.870 ± 0.016	0.0126 ± 0.0021	0.9956	0.0086 ± 0.0006	101 ± 0.6	0.7506 ± 0.0298	0.9957
YGC	0.649 ± 0.043	0.0085 ± 0.0007	0.9982	0.0086 ± 0.0002	102 ± 2.0	0.7273 ± 0.0067	0.9951
YMA	0.901±0.032	0.0126 ± 0.0016	0.9959	0.0088 ± 0.0001	87 ± 1.0	0.7731 ± 0.0006	0.9962

**Table 3:** MIC of the *F. pinicola* extracts against pathogenic bacteria, µg/mL.

Test Microorganism	Water	Methanol	Ethanol	Butanol	Ethyl Acetate	Methylene Chloride	Hexane	Hot Water
*Staphylococcus aureus* ATCC 25923	1250	1250	1250	1250	2500	2500	1250	1250
*Listeria monocytogenes* ATCC 8787	1250	2500	2500	1250	2500	625	1250	1250
*Bacillus subtilis* ATCC 6633	1250	625	625	625	625	625	625	1250
*Bacillus cereus* ATCC 11778	1250	625	1250	1250	625	1250	1250	1250
*Pseudomonas aeruginosa* ATCC 9027	625	625	625	625	2500	625	1250	1250
*Klebsiella pneumoniae* ATCC 13883	625	625	625	625	625	625	625	1250
*Escherichia coli* ATCC 8739	312.5	625	625	625	2500	625	1250	312.5
*Salmonella enterica* ssp. *enterica* ser. Enetritidis ATCC 13076	625	625	625	1250	2500	625	312.5	625

## Data Availability

The original contributions presented in this study are included in the article. Further inquiries can be directed to the corresponding author.

## Abbreviations

The following abbreviations are used in this manuscript:

RBCA	Rose Bengal Chloramphenicol Agar
MCM	Mushroom Complete Medium
UPGMA	Unweighted Pair Group Method using the Arithmetic Average
CD	Czapek Dox
GP	Glucose-peptone
MEA	Malt extract agar
MYE	Malt yeast extract
PDA	Potato dextrose agar
YEA	Yeast extract agar
YGC	Yeast glucose chloramphenicol
YMA	Yeast extract malt extract agar
DW	Dry weight
MIC	Minimum Inhibitory Concentration
SD	Standard deviation
ANOVA	Analysis of Variance

## References

[1] Fu C., Song Y., Zhang D., Wang H., Chen X., Li J. (2024). Whole genome sequencing and analysis of *Inonotus hispidus* isolated from the Chengde Mountain Resort, China. Res. Sq..

[2] Wu J., Yang X., Duan Y., Wang P., Qi J., Gao J.-M., Liu C. (2022). Biosynthesis of sesquiterpenes in basidiomycetes: A review. J. Fungi.

[3] Halbwachs H., Harper C.J., Krings M. (2021). Fossil Ascomycota and Basidiomycota, with notes on fossil lichens and nematophytes. Encycl. Mycol..

[4] Ghobad-Nejhad M., Dima B., Cui B.-K., Si J. (2023). Editorial: Basidiomycete fungi: From biosystematics and biodiversity to biotechnology. Front. Microbiol..

[5] Sułkowska-Ziaja K., Szewczyk A., Galanty A., Gdula-Argasińska J., Muszyńska B. (2018). Chemical composition and biological activity of extracts from fruiting bodies and mycelial cultures of *Fomitopsis betulina*. Mol. Biol. Rep..

[6] Sandargo B., Chepkirui C., Cheng T., Chaverra-Muñoz L., Thongbai B., Stadler M., Hüttel S. (2019). Biological and chemical diversity go hand in hand: Basidiomycota as source of new pharmaceuticals and agrochemicals. Biotechnol. Adv..

[7] Toju H., Tanabe A.S., Yamamoto S., Sato H. (2012). High-coverage ITS primers for the DNA-based identification of ascomycetes and basidiomycetes in environmental samples. PLoS ONE.

[8] Mowna Sundari T., Alwin Prem Anand A., Jenifer P., Shenbagarathai R. (2018). Bioprospection of Basidiomycetes and molecular phylogenetic analysis using internal transcribed spacer (ITS) and 5.8S rRNA gene sequence. Sci. Rep..

[9] Hillis D.M. (1997). Phylogenetic analysis. Curr. Biol..

[10] Bishop K.S. (2020). Characterisation of extracts and anti-cancer activities of *Fomitopsis pinicola*. Nutrients.

[11] Liu S., Song C.G., Xu T.M., Ji X., Wu D.M., Cui B.K. (2022). Species diversity, molecular phylogeny, and ecological habits of *Fomitopsis* (Polyporales, Basidiomycota). Front. Microbiol..

[12] Han M.L., Chen Y.Y., Shen L.L., Song J., Vlasák J., Dai Y.C., Cui B.K. (2016). Taxonomy and phylogeny of the brown-rot fungi: *Fomitopsis* and its related genera. Fungal Divers..

[13] Liu S., Han M.L., Xu T.M., Wang Y., Wu D.M., Cui B.K. (2021). Taxonomy and phylogeny of the *Fomitopsis pinicola* complex with descriptions of six new species from East Asia. Front. Microbiol..

[14] Sigoillot J.C., Berrin J.G., Bey M., Lesage-Meessen L., Levasseur A., Lomascolo A., Record E., Uzan-Boukhris E. (2012). Fungal Strategies for Lignin Degradation. Adv. Bot. Res..

[15] Varghese R., Dalvi Y.B., Lamrood P.Y., Shinde B.P., Nair C.K.K. (2019). Historical and current perspectives on therapeutic potential of higher basidiomycetes: An overview. 3 Biotech.

[16] Wainwright M. (2008). Some highlights in the history of fungi in medicine—A personal journey. Fungal Biol. Rev..

[17] Kurchenko V.P., Sushinskaya N.V., Kiseleva I.S., Ermoshin A.A. (2022). Biologically active substances in fruit bodies of wood decomposing fungi. Proceedings of the Actual Problems of Organic Chemistry and Biotechnology (OCBT2020): Proceedings of the International Scientific Conference.

[18] Zahid T.M., Idrees M., Ying W., Zaki H.A., Abdullah I., Haiying B. (2020). Review of chemical constituents and pharmacology of brown-rot fungus Fomitopsis pinicola. J. Nat. Sci. Res..

[19] Wang Y., Cheng X., Wang P., Wang L., Fan J., Wang X., Liu Q. (2014). Investigating migration inhibition and apoptotic effects of *Fomitopsis pinicola* chloroform extract on human colorectal cancer SW-480 cells. PLoS ONE.

[20] Cheng J.J., Lin C.Y., Lur H.S., Chen H.P., Lu M.K. (2008). Properties and biological functions of polysaccharides and ethanolic extracts isolated from medicinal fungus, *Fomitopsis pinicola*. Process Biochem..

[21] Ravikumar K.S., Ramya H., Ajith T.A., Shah M.A., Janardhanan K.K. (2021). Bioactive extract of *Fomitopsis pinicola* rich in 11-α-acetoxykhivorin mediates anticancer activity by cytotoxicity, induction of apoptosis, inhibition of tumor growth, angiogenesis and cell cycle progression. J. Funct. Foods.

[22] Wu X., Wu Y., Ye L., Wu L., Su C., Iyu X., Fu J. (2023). The protective effect and potential mechanism analysis of Fomitopsis pinicola mycelia polysaccharides (FPMPS) on acute alcoholic liver injury in mice. 2023, preprint. Authorea.

[23] Taylor T.N., Krings M., Taylor E.L. (2015). Basidiomycota. Foss. Fungi.

[24] Bakratsas G., Polydera A., Katapodis P., Stamatis H. (2021). Recent trends in submerged cultivation of mushrooms and their application as a source of nutraceuticals and food additives. Future Foods.

[25] Krupodorova T., Barshteyn V., Sekan A. (2021). Review of the basic cultivation conditions influence on the growth of basidiomycetes. Curr. Res. Environ. Appl. Mycol..

[26] Tang Y.J., Zhu L.W., Li H.M., Li D.S. (2007). Submerged culture of mushrooms in bioreactors—Challenges, current state-of-the-art, and future prospects. Food Technol. Biotechnol..

[27] Angelova G., Stefanova P., Brazkova M., Krastanov A. (2023). Molecular and morphological characterization of *Xylaria karsticola* (Ascomycota) isolated from the fruiting body of *Macrolepiota procera* (Basidiomycota) from Bulgaria. PLoS ONE.

[28] Stefanova P., Brazkova M., Angelova G. (2022). Comparative study of DNA extraction methods for identification of medicinal mushrooms. BIO Web Conf..

[29] Altschul S.F., Gish W., Miller W., Myers E.W., Lipman D.J. (1990). Basic local alignment search tool. J. Mol. Biol..

[30] Gene Bank Database. https://www.ncbi.nlm.nih.gov.

[31] QIAGEN Digital Insights. https://digitalinsights.qiagen.com.

[32] Saitou N., Nei M. (1987). The neighbor-joining method: A new method for reconstructing phylogenetic trees. Mol. Biol. Evol..

[33] Jo W.S., Kim D.G., Seok S.J., Jung H.Y., Park S.C. (2014). The culture conditions for the mycelial growth of *Auricularia auricula-judae*. J. Mushrooms.

[34] Bouguettoucha A., Balannec B., Amrane A. (2011). Unstructured models for lactic acid fermentation—A review. Food Technol. Biotechnol..

[35] Ibarz A., Augusto P.E. (2015). An autocatalytic kinetic model for describing microbial growth during fermentation. Bioprocess Biosyst. Eng..

[36] Choi M., Al-Zahrani S.M., Lee S.Y. (2014). Kinetic model-based feed-forward controlled fed-batch fermentation of *Lactobacillus rhamnosus* for the production of lactic acid from Arabic date juice. Bioprocess Biosyst. Eng..

[37] Kemmer G., Keller S. (2010). Nonlinear least-squares data fitting in Excel spreadsheets. Nat. Protoc..

[38] Angelova G., Brazkova M., Mihaylova D., Slavov A., Petkova N., Blazheva D., Deseva I., Gotova I., Dimitrov Z., Krastanov A. (2022). Bioactivity of Biomass and Crude Exopolysaccharides Obtained by Controlled Submerged Cultivation of Medicinal Mushroom *Trametes versicolor*. J. Fungi.

[39] CLSI (2012). Methods for Dilution Antimicrobial Susceptibility Tests for Bacteria That Grow Aerobically, Approved Standard.

[40] Bower J.A. (1998). Statistics for food science V: ANOVA and multiple comparisons (Part B). Nutr. Food Sci..

[41] Gáper J., Gáperová S., Pristaš P., Šebesta M., Kollárová P., Gallay I., Slobodník B. (2025). The geographical distribution, trophic modes, and host preferences of *Fomitopsis pinicola* in Central Europe: A comprehensive review. Cent. Eur. For. J..

[42] Dresch P., D’Aguanno M.N., Rosam K., Grienke U., Rollinger J.M., Peintner U. (2015). Fungal strain matters: Colony growth and bioactivity of the European medicinal polypores *Fomes fomentarius, Fomitopsis pinicola* and *Piptoporus betulinus*. AMB Express.

[43] Gaude N., Bortfeld S., Erban A., Kopka J., Krajinski F. (2015). Symbiosis-dependent accumulation of primary metabolites in arbuscule-containing cells. BMC Plant Biol..

[44] Pekşen A., Kibar B. (2016). Effects of various carbon and nitrogen sources on mycelial biomass production of *Macrolepiota procera* and *Polyporus squamosus* in submerged culture. Anadolu Tarım Bilim. Derg..

[45] Wiriya J., Kavinlertvatana P., Lumyong S. (2014). Effects of different culture media, carbon and nitrogen sources and solid substrates on growth of Termitomyces mushrooms. Chiang Mai J. Sci..

[46] Bellettini M.B., Fiorda F.A., Maieves H.A., Teixeira G.L., Ávila S., Hornung P.S., Júnior A.M., Ribani R.H. (2019). Factors affecting mushroom *Pleurotus* spp.. Saudi J. Biol. Sci..

[47] Abdel Aziz N.H., Yousef N.S., El-Haddad M.E., El-Tayeb T.S. (2018). Influence of nutritional and climatic conditions on mycelial growth of three oyster mushroom strains. Arab Univ. J. Agric. Sci..

[48] Arana-Gabriel Y., Burrola-Aguilar C., Alcala-Adan A., Zepeda-Gomez C., Estrada Zuniga M.E. (2020). Mycelial growth of the edible wild mushroom *Floccularia luteovirens* in different culture mediums and pH. Agro Prod..

[49] Muthu N., Shanmugasundaram K. (2015). Effect of five different culture media on mycelial growth of Agrocybe aegerita. Int. J. Pharm. Sci. Res..

[50] Gbolagade J.S., Fasidi I.O., Ajayi E.J., Sobowale A.A. (2006). Effect of physico-chemical factors and semi-synthetic media on vegetative growth of *Lentinus subnudus* (Berk.), an edible mushroom from Nigeria. Food Chem..

[51] Alam N., Shim M.J., Lee M.W., Shin P.G., Yoo Y.B., Lee T.S. (2009). Vegetative growth and phylogenetic relationship of commercially cultivated strains of Pleurotus eryngii based on ITS sequence and RAPD. Mycobiology.

[52] Kim S.W., Hwang H.J., Park J.P., Cho Y.J., Song C.H., Yun J.W. (2002). Mycelial growth and exo-biopolymer production by submerged culture of various edible mushrooms under different media. Lett. Appl. Microbiol..

[53] Fletcher I., Freer A., Ahmed A., Fitzgerald P. (2019). Effect of temperature and growth media on mycelium growth of *Pleurotus ostreatus* and *Ganoderma lucidum* strains. Cohes. J. Microbiol. Infect. Dis..

[54] Jo W.S., Kang M.G., Choi S.Y., Yoo Y.B., Seok S.J., Jung H.Y. (2010). Culture conditions for mycelial growth of *Coriolus versicolor*. Mycobiology.

[55] Jo W.S., Cho Y.J., Cho D.H., Park S.D., Yoo Y.B., Seok S.J. (2009). Culture conditions for the mycelial growth of *Ganoderma applanatum*. Mycobiology.

[56] Liu X.T., Winkler A.L., Schwan W.R., Volk T.J., Rott M., Monte A. (2010). Antibacterial Compounds from Mushrooms II: Lanostane Triterpenoids and an Ergostane Steroid With Activity Against *Bacillus cereus* Isolated from *Fomitopsis Pinicola*. Planta Medica.

[57] Keller A.C., Maillard M.P., Hostettmann K. (1996). Antimicrobial Steroids from the Fungus *Fomitopsis Pinicola*. Phytochemistry.

[58] Karaca B., Kyalo Kilonzo N., Korkmaz Ş., Onar O., Yıldırım Ö., Çöleri Cihan A. (2025). The Dual Role of the Medicinal Mushroom *Fomitopsis pinicola* in Inhibiting Biofilm and Reducing Antibiotic Resistance of Methicillin-Resistant *Staphylococcus aureus*. Food Sci. Nutr..

[59] Pala S.A., Wani A.H., Ganai B.A. (2019). Antimicrobial Potential of Some Wild Macromycetes Collected from Kashmir Himalayas. Plant Sci. Today.

[60] Huguet C., Bourjot M., Bellanger J.M., Prévost G., Urbain A. (2022). Screening for Antibacterial Activity of French Mushrooms Against Pathogenic and Multidrug Resistant Bacteria. Appl. Sci..

[61] Bragina O., Kuhtinskaja M., Elisashvili V., Asatiani M., Kulp M. (2025). Antibacterial Properties of Submerged Cultivated Fomitopsis pinicola, Targeting Gram-Negative Pathogens, Including Borrelia burgdorferi. Sci..

[62] Alves M.J., Ferreira I.C., Martins A., Pintado M. (2012). Antimicrobial Activity of Wild Mushroom Extracts Against Clinical Isolates Resistant To Different Antibiotics. J. Appl. Microbiol..

[63] Gebreyohannes G., Nyerere A., Bii C., Berhe Sbhatu D. (2019). Determination of Antimicrobial Activity of Extracts of Indigenous Wild Mushrooms against Pathogenic Organisms. eCAM.

